# Common Bean Fe Biofortification Using Model Species' Lessons

**DOI:** 10.3389/fpls.2017.02187

**Published:** 2017-12-22

**Authors:** Raul A. Sperotto, Felipe K. Ricachenevsky

**Affiliations:** ^1^Biological Sciences and Health Center, Graduate Program in Biotechnology, University of Taquari Valley - UNIVATES, Lajeado, Brazil; ^2^Graduate Program in Agrobiology, Biology Department, Federal University of Santa Maria, Santa Maria, Brazil; ^3^Graduate Program in Cell and Molecular Biology, Federal University of Rio Grande do Sul, Porto Alegre, Brazil

**Keywords:** anti-nutrient, bean, biofortification, iron, model species, *Phaseolus vulgaris*, transgenic strategies

Common bean (*Phaseolus vulgaris* L.) is the most widely grown grain legume for direct human consumption and is highly preferred in many parts of Africa and Latin America, as well as in southern Europe (Broughton et al., 2003; Blair and Izquierdo, 2012). It is an important source of nutrients for more than 300 million people, representing 65% of total protein consumed, 32% of energy, and a major source of micronutrients e.g., iron (Fe), zinc, thiamin, and folic acid (Welch et al., 2000; Broughton et al., 2003; Blair et al., 2010a; Petry et al., 2015). It is known as the “poor men's meat,” due to its high protein, minerals, and vitamins content (Blair, 2013). Fe is an essential micronutrient for almost all living organisms (Bashir et al., 2013), and Fe deficiency is the most common micronutrient deficiency worldwide, disproportionately affecting the poorest and most vulnerable populations in resource-limited settings, leading to Fe deficiency anemia (IDA; Stevens et al., 2013; Finkelstein et al., 2017). IDA is difficult to address through Fe supplementation or processed foods; therefore, several attempts are being made to enhance Fe accumulation into staples such as rice, maize, wheat, and legumes (Blair and Izquierdo, 2012) using biofortification, which is the process of breeding or genetic engineering to improve nutrient content in a crop (Blair, 2013). Biofortification is considered a sustainable and cost effective strategy to address malnutrition in developing countries because it targets staple foods that are consumed daily (Dwivedi et al., 2012).

Nutritional quality in common beans has been found to be higher than in cereals, with large amounts of minerals and vitamins accumulated in the seeds (Broughton et al., 2003). Common bean is estimated to have 4–10 times the amount of Fe, and 2–3 times the amount of Zn compared to rice (Pfeiffer and McClafferty, 2007). Also, these minerals and vitamins are generally retained after harvest and processing, while in polished cereal grains the Fe-rich tissues (embryo and aleurone layer) are lost (Wang et al., 2003). Although the average Fe concentration in beans is high, many people still suffer from IDA due to an insufficient level of bioavailable Fe in a monotonous cereal/bean-based diet without meat (Bouis, 2007). For Fe biofortification purposes, the use of common bean is advantageous because the baseline grain Fe content is high at 55 ppm and variability for the trait is great (Petry et al., 2015), ranging up to 110 ppm, allowing initial biofortification attempts to start from already high Fe levels (Blair et al., 2012; Blair, 2013). Another advantage of using common beans in biofortification programs is that seeds are consumed whole after boiling. Therefore, all major components of the common bean seed could be targets of biofortification: seed coat, cotyledons, and embryo (Blair et al., 2013).

The target Fe level of HarvestPlus, an international research program supporting the research and development of biofortified crops, is 94 ppm in whole bean seeds (Blair and Izquierdo, 2012; Petry et al., 2015). According to Vasconcelos et al. (2017), in order to achieve 30% of the estimated average daily dietary requirement for Fe on a dry weight (DW) basis, the recommended Fe levels in whole beans should be 107 ppm. The target level was quickly reached, and in some countries plant breeders have already developed and released new *P. vulgaris* bean varieties with Fe concentrations of about 100 ppm (Petry et al., 2015). These varieties show good micronutrient retention after processing, and equal or increased agronomic yield (Bouis and Welch, 2010). However, successful bean Fe biofortification might be constrained due to the reported low Fe bioavailability (Ariza-Nieto et al., 2007) associated with high concentrations of Fe absorption inhibitors, also called anti-nutrients, such as polyphenols and phytate (Beninger et al., 2005; Petry et al., 2014). Here we propose multiple, complementary approaches to increase Fe concentration and bioavailability in common bean, based on the current knowledge on model species. These approaches are summarized in Figure 1.

**Figure 1:**
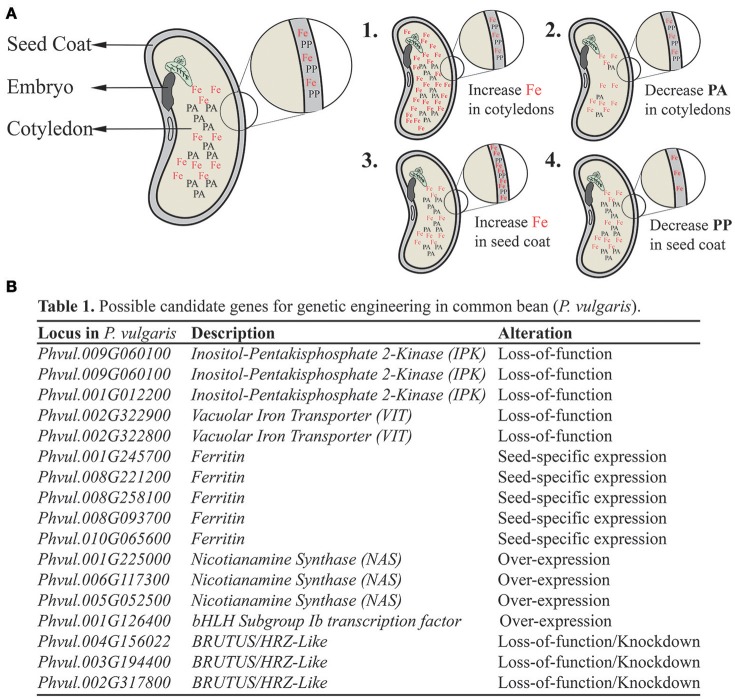
Summary of proposed strategies to increase bioavailable Fe delivery in common bean (*Phaseolus vulgaris*) seeds. Different strategies might be used as complementary, non-excludent approaches for bean biofortification. **(A)** Schematic representation of common bean seed and its main parts: seed coat, embryo and cotyledons. In cotyledons, iron (Fe) is shown with phytate (PA), whereas in the seed coat (detail), Fe is shown with polyphenols (PP). Each one act as an Fe absorption inhibitor in the human gut, with PA being likely a stronger anti-nutrient than PP. (1) Strategy aiming at increasing Fe concentration in the cotyledons to overcome PA anti-nutrient properties. (2) Strategy aiming at decreasing PA concentration in the cotyledons, making Fe in this tissue more bioavailable. (3) Strategy aiming at increasing Fe concentration in the seed coat to overcome PP anti-nutrient properties. (4) Strategy aiming at decreasing PP concentration in the seed coat, making Fe in this tissue more bioavailable. **(B)** Proposed candidate genes for genetic engineering in common bean, based on previous studies in model species. These genes are orthologous to genes found in *Arabidopsis thaliana* based on BLAST searches, except for Phvul.001G012200, which is the best hit using a soybean (*Glycine max*) *IPK* gene as query (Yuan et al., 2012). For each candidate gene, the type of manipulation is indicated.

## Decreasing anti-nutrient concentration and co-localization with Fe in seeds

Short-term human isotope studies indicate that phytate is the major Fe absorption inhibitor in beans, with polyphenols playing a minor role (Petry et al., 2012, 2014). Phytate increases with the Fe concentration in beans, and both are mainly found in the cotyledons. It should be possible to simultaneously breed for high Fe and low phytate since most phytate-related QTLs are independent of Fe concentration QTLs (Blair et al., 2012, 2013). Two main strategies for phytate reduction in seeds have been attempted: disruption of its biosynthetic pathway with knockout/knockdown of *inositol pentakisphosphate 2-kinase* (*IPK1*) in Arabidopsis and rice showing decreased phytate in seeds and normal yield (Stevenson-Paulik et al., 2005; Ali et al., 2013), but with possible defects in Pi homeostasis (Kuo et al., 2014); and mutations on phytate vacuolar transporters expressed in seeds, which reduced phytate concentration in other species (Shi et al., 2007; Nagy et al., 2009; Xu et al., 2009). In common bean, disruption of the orthologous transporter *PvMRP6* resulted in 90% less phytate in seeds and normal agronomic performance (Panzeri et al., 2011; Campion et al., 2013). However, seeds were hard to cook and induced digestive problems in human subjects (Petry et al., 2016). Thus, further research is necessary to improve Fe bioavailability by decreasing phytate while maintaining agronomic performance and consumer preferences.

Biofortification in beans can target all seed tissues: the thick seed coat, two large cotyledons and a well-developed embryo (Blair et al., 2013), which comprise 7–10, 85, and 2–3% of seed weight, respectively (Ariza-Nieto et al., 2007). Remarkably, segregating populations derived from crosses between wild and cultivated parents showed that QTLs for Fe accumulation in each tissue segregate separately, and the Fe range and maximum amount observed in seed coat is higher than in cotyledons (Blair et al., 2013). Seed coat can contribute with as much as 26% of the total seed Fe, and polyphenols, not phytate, are the main anti-nutrients in the tissue (Ariza-Nieto et al., 2007). Thus, exploring seed coat biofortification is promising, as little is known about which specific polyphenol molecules reduce Fe bioavailability and how reduction in their concentration might affect plant and seed physiology (Petry et al., 2015).

## Further increasing Fe accumulation in beans

Genetic engineering beans to accumulate higher Fe concentrations in seeds can benefit from work on model plants. Vacuolar Iron Transporter (VIT) family members are likely candidates, since they are involved in seed Fe localization and/or concentration in Arabidopsis and rice (Kim et al., 2006; Zhang et al., 2012). AtVIT1 localizes Fe to the provasculature, and changes in provasculature density have been proposed as a means to increase Fe content in seeds (Roschzttardtz et al., 2017). In rice, OsVIT1 and OsVIT2 are involved in flag leaf Fe pool regulation, and might also have a role in seed Fe localization (Zhang et al., 2012). Recent work showed that endosperm-specific overexpression of *TaVIT2* increased Fe concentration in wheat endosperm (Connorton et al., 2017), indicating that *VIT* genes can increase tissue Fe sink strength.

In rice, overexpression of *NICOTIANAMINE SYNTHASE* (*NAS*) genes was shown to substantially increase Fe concentration in the endosperm, presumably increasing Fe translocation through the phloem (Johnson et al., 2011). In addition, *OsNAS1* over-expression in rice plants enhance human Fe bioavailability from the high nicotianamine (NA) grains (Zheng et al., 2010). NA role in Fe long-distance transport is likely to be conserved in land plants (Schuler and Bauer, 2011), and therefore a similar approach could be applied to common bean. Wirth et al. (2009) overexpressed bean *Ferritin*, Arabidopsis *Nicotianamine synthase*, and *Aspergillus fumigatus Phytase* genes in rice plants, and detected 6.3-fold increase in Fe concentration on the polished seeds. Aluru et al. (2011) used a *lpa* maize mutant to overexpress soybean *Ferritin* gene, and found 2.7-fold increase in seed Fe concentration. Similar approaches could be certainly used in common bean plants in order to concomitantly decrease phytate levels and increase Fe accumulation and availability.

Another approach would be to explore genes that regulate Fe concentration. Over-expression of *AtbHLH039* results in constitutive Fe deficiency response and increased Fe levels in leaves and seeds (Naranjo-Arcos et al., 2017). Interestingly, the bean genome has only one gene similar to all four subgroup Ib from Arabidopsis, which are known to be involved in Fe deficiency response (Brumbarova et al., 2015). Work in Arabidopsis and rice has shown that the negative regulators of Fe deficiency response BRUTUS/HRZ-like proteins could lead to increased Fe concentration in seeds of knockout/knockdown plants (Kobayashi et al., 2013; Hindt et al., 2017). Three *BRUTUS/HRZ-like* genes are found in the bean genome. Although promising, manipulation of regulatory proteins should be performed with caution, since plants might display undesired phenotypic changes besides increased Fe in seeds.

Common bean genetic transformation protocols are lengthy and of low reproducibility, while *in vitro* plant regeneration is especially difficult (Veltcheva et al., 2005; Rech et al., 2008). The *Agrobacterium rhizogenes* system allow for bean root transformation and could be used for characterization and selection of candidate genes for stable transformation (Estrada-Navarrete et al., 2007). Another solution is CRISPR-Cas9-mediated genome editing, which does not necessarily require transformation (Malnoy et al., 2016; Wolt et al., 2016) and could circumvent the problem in the near future. However, using this method, it would be easier to knockout a specific gene than overexpress it.

## Exploring bean natural variation and wild relatives

The wide genetic Fe variability of beans has enabled plant breeders to develop varieties with twice Fe compared to normal beans (Blair et al., 2010b). Common bean is native to Latin America, and is one of the five cultivated species of the *Phaseolus* genus. It has two main genetic pools: Andean (large seeds) and Mesoamerican (small seeds). Andean and inter-gene-pool hybrids have higher Fe concentrations compared to Measoamerican ones, although the range of variation is similar (Blair, 2013). Large germplasm collection screenings for high Fe genotypes conducted in local and wild varieties of *P. vulgaris* have reported up to 110 ppm Fe. However, early analyses on closely related species such as *P. coccineus* and *P. dumosus* have found up to 127 ppm Fe, indicating that wild relatives might be useful (Blair et al., 2013). Even considering that high Fe wild genetic material showed poor agronomical performance (and introgression might not be straightforward in interspecific crosses), further screening of wild genotypes is promising. Moreover, wild beans accumulate more Fe in seed coats and less in cotyledons compared to domesticated genotypes, indicating that they can contribute differently for tissue-specific biofortification (Blair et al., 2013).

QTL studies show that multiple genes regulate seed Fe levels (Blair and Izquierdo, 2012; Blair et al., 2013). Interestingly, Fe concentration inheritance seems to be associated with Zn concentration, as found in other crops, indicating that similar genes are involved in micronutrient loading in seeds and that breeding for both minerals simultaneously is feasible (Blair et al., 2013). Based on QTL localization, Fe and Zn concentration might be associated with the seed storage protein Phaseolin, whereas the Fe storage protein Ferritin was also associated with a QTL (Blair et al., 2009). Indeed, engineering for increased Ferritin expression in endosperm of Poaceae species have been a relatively successful strategy (Sperotto et al., 2012), and thus Ferritin-associated QTLs are interesting candidates. Fe-chelate reductase, which is important for Fe uptake in roots, has also been suggested as a possible candidate gene (Blair et al., 2013).

## Where to focus next?

Biofortification for any crop will benefit from multiple approaches, which can improve one another to achieve target Fe seed levels. For common bean, bioavailability tests are key due to the high level of anti-nutrients. The Caco-2 cell *in vitro* model has been widely used, with better results than previous *in vivo* absorption models (Ariza-Nieto et al., 2007; Blair et al., 2013; Petry et al., 2016). Recently, a new model using poultry (*Gallus gallus*) combined with Caco-2 cells showed that they can be used as a robust, cost-effective two-step system to evaluate Fe bioavailability, which should be mandatory to generate as well as to monitor biofortified crop seeds after their release (Tako et al., 2016).

Another focus should be to independently increase Fe in cotyledons and in seed coat, and understand the physiological roles of phytate/polyphenols and the effects of their reduction on seed viability and seedling establishment. Fe in cotyledons accumulates at the vascular bundles (Cvitanich et al., 2010), similar to Arabidopsis where it depends on Vacuolar Iron Transporter (VIT1; Kim et al., 2006). Phytate is also likely to accumulate in vacuoles, based on the activity of MRP transporters (Nagy et al., 2009; Panzeri et al., 2011). It remains to be determined if the same cells accumulate Fe and phytate, and if the vacuole is the main site where phytate-bound Fe is localized. Thus, analyses of cellular and sub-cellular distribution of Fe and phytate (using phosphorous as a surrogate) will be key for advances in cotyledon biofortification (Punshon et al., 2013). Moreover, understanding how polyphenols affect Fe homeostasis and how their levels could be manipulated will indicate new approaches for seed coat biofortification.

## Author contributions

All authors listed have made a substantial, direct and intellectual contribution to the work, and approved it for publication.

### Conflict of interest statement

The authors declare that the research was conducted in the absence of any commercial or financial relationships that could be construed as a potential conflict of interest.
